# Allokation von einmalig zu applizierenden Arzneimitteln bei Kindern in globalen Compassionate Use-Programmen

**DOI:** 10.1007/s00481-022-00722-w

**Published:** 2022-09-30

**Authors:** Clemens Miller

**Affiliations:** Klinik für Anästhesiologie, Universitätsmedizin Göttingen, Robert-Koch-Straße 40, 37075 Göttingen, Deutschland

**Keywords:** Allokation, Compassionate Use, Gentherapie, Härtefall, Kinder, Onasemnogenum abeparvovecum, Verteilungsgerechtigkeit, Allocation, Compassionate use, Distribution justice, Expanded access, Gene therapy, Pediatrics, Onasemnogenum abeparvovecum

## Abstract

Compassionate Use beschreibt die Anwendung zulassungsüberschreitender Arzneimittel für Patient*innengruppen, die an einer lebensbedrohlichen oder zu einer schweren Behinderung führenden Erkrankung leiden, ohne dass eine alternative Therapieoption besteht. An Ärzt*innen vorbei werden solche Programme ausschließlich von Pharmaunternehmen initiiert, was viele ethische Konflikte mit sich bringt. Eine neue Dimension erreichte das 2020 gestartete Programm für *Onasemnogenum abeparvovecum* zur Therapie von Spinaler Muskelatrophie bei Kindern, welches die Krankheit nach nur einmaliger Gabe stoppen sollte. Die globale Allokation von nur 100 zur Verfügung gestellten Dosierungen per Losverfahren stellte ein Novum bei der Allokation in Compassionate Use-Programmen dar und wurde vielfach kritisiert. Diese Arbeit untersucht mögliche alternative Allokationsprinzipien auf das Beispiel von *Onasemnogenum abeparvovecum*. Für jedes in Frage kommende Prinzip der Allokation medizinischer Güter bestehen Aspekte, die im Hinblick auf die drängende Zeit und die globale Verteilung bedacht werden müssen. Dies lässt einige Prinzipien wie First-Come-First-Served vernachlässigbar erscheinen. Verbliebene Prinzipien werden hierarchisch geordnet, um einen Algorithmus abzuleiten, der eine Alternative zum Losverfahren darstellen kann. Eine Kombination von Teilnahmebereitschaft bei Forschung, Dringlichkeit und Erfolgsaussicht (bezogen auf die Existenz supportiver Therapieoptionen) kann in ähnlichen Fällen bei zukünftigen globalen Compassionate Use-Programmen bei Kindern erwogen werden. Da universelle Algorithmen nur schwer definierbar sind, sollten Allokationskriterien in jedem Fall durch ein unabhängiges Expert*innengremium diskutiert werden. Sowohl die Konstitution eines solchen Gremiums sowie deren verpflichtende Konsultierung sind gefordert, um für Entlastung aller Beteiligten zu sorgen und Willkür vorzubeugen.

## Abstract (en)

**Definition of the problem:**

Compassionate use is the use of unapproved drugs in groups of patients suffering from a disease that, in the absence of an alternative treatment option, is life-threatening or leads to severe disability. Physicians are not in charge because access to the drug is only granted by pharmaceutical companies, which comes along with many ethical issues. Launched in 2020, the program of *Onasemnogenum abeparvovecum* against spinal muscular atrophy in children reached a new dimension. The intent of this drug is to stop the progression of the disease with just a single dose, but the company limited the doses in the program to only 100. The global allocation was by lottery, which was considered a novelty in compassionate use history and therefore widely criticized. This paper investigates alternative allocation principles.

**Arguments:**

Each possible principle is accompanied by many aspects that need to be considered with regard to urgency and global distribution. This makes some principles like first-come-first-served seem negligible. Remaining principles are ordered hierarchically to derive an algorithm that can represent an alternative to a lottery. A combination of willingness to participate in research, urgency, and likelihood of success (relative to the availability of supportive treatment options) may be considered in similar cases in future global compassionate use programs with children.

**Conclusion:**

Since universal algorithms are difficult to define, allocation criteria should always be discussed by an independent panel of experts. Both the constitution of such a panel and its mandatory consultation are necessary in order to decrease the burden for all those involved and to prevent arbitrariness.

## Compassionate Use-Programme

„Compassionate Use“ (im Deutschen auch Härtefall genannt) bezeichnet die Anwendung von bisher nicht zugelassenen Arzneimitteln für Patient*innengruppen außerhalb klinischer Studien. Aufgrund vielversprechender Resultate klinischer Studien steht die indikationsbezogene Zulassung für ein solches Arzneimittel jedoch in Kürze bevor oder ist bereits beantragt. Als eine von drei in Deutschland praxisrelevanten Formen der zulassungsüberschreitenden Arzneimittelanwendung ist Compassionate Use abzugrenzen von „Off-label Use“ (Anwendung eines Arzneimittels für eine andere Indikation als die zugelassene) und dem individuellen Heilversuch (Anwendung nur für einzelne Patient*innen).

In den letzten Jahren fand Compassionate Use zunehmend Anwendung. Grund dafür ist die lange Zeitspanne zwischen dem Abschluss einer klinischen Phase-III-Studie mit vielversprechenden Resultaten und der schlussendlichen Zulassung dieses Arzneimittels, was mitunter Jahre dauern kann. Auch wenn durch zahlreiche Förderprogramme – wie z. B. einer beschleunigten Zulassung – die Dauer der Zulassungsverfahren in den letzten Jahren teilweise deutlich verkürzt werden konnte (Blasius 2014), benötigen die Behörden in den USA und Europa dennoch ungefähr ein Jahr für den Zulassungsprozess (Downing et al. 2017).

**Figure 1:**
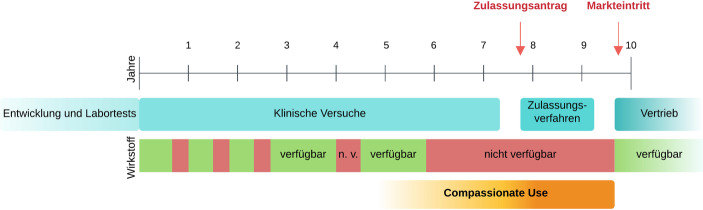

Für Betroffene von beispielsweise onkologischen oder neurodegenerativen Erkrankungen kann dies eine sehr bzw. zu lange Zeitspanne sein, wenn sie zuvor in einer der klinischen Studien eingeschlossen waren und auf die kontinuierliche Einnahme dieses Arzneimittels angewiesen sind. Wiederum andere Patient*innen könnten ebenso von diesem Arzneimittel profitieren, konnten jedoch gar nicht in eine der klinischen Studien aufgenommen werden – beispielsweise weil die Einschluss- und Ausschlusskriterien zu spezifisch waren, die Rekrutierung der berechneten Fallzahl schon abgeschlossen war, oder die Studie an Vorgaben oder Örtlichkeiten gebunden war, welche die Patient*innen nicht erfüllen konnten (Meier 2016). Daher gibt es im Zeitraum bis zur Zulassung eines Arzneimittels aus Patient*innensicht immer wieder Versorgungslücken, wie Abb. 1 schematisch darstellt. Bedarf für Compassionate Use-Programme besteht vor allem im Zeitraum zwischen Zulassungsantrag und Markteintritt, aber gelegentlich auch schon davor, z. B. nach Abschluss der Rekrutierung zur Zulassungsstudie.

Für solche Härtefälle wurde deshalb durch die nach Umsetzung der europäischen Direktive *EG 726/2004 Art. 83* 2009 in Kraft getretene *Verordnung über das Inverkehrbringen von Arzneimitteln ohne Genehmigung oder ohne Zulassung in Härtefällen *geregelt, dass Betroffenen, die an einer zu einer schweren Behinderung führenden oder lebensbedrohlichen Erkrankung leiden, Arzneimittel zur Verfügung gestellt werden können, wenn es bereits ausreichend Hinweise auf deren klinische Wirksamkeit und Sicherheit gibt und es an Alternativen mangelt. Anders als beim „Off-label Use“ und beim individuellen Heilversuch obliegt die Letztentscheidung über die Anordnung des Arzneimittels jedoch nicht, wie sonst in der Medizin üblich, bei den behandelnden Ärzt*innen im Konsens mit deren Patient*innen, sondern einzig bei dem herstellenden Pharmaunternehmen. Dass die Entscheidungshoheit bei dem herstellenden Pharmaunternehmen liegt, ist nicht nur in Deutschland so, sondern vergleichbar mit den meisten Staaten mit Compassionate Use-Bestimmungen (Caplan und Ray 2016).

Ein Pharmaunternehmen kann selbständig entscheiden, ein spezielles Programm zu initiieren, in welchem hergestellte Arzneimittel bereits vor deren behördlicher Zulassung an Bedürftige abgegeben werden. In der Verordnung festgelegte Voraussetzungen zum Start eines Compassionate Use-Programms sind 1) die Alternativlosigkeit des Arzneimittels, 2) die kostenlose Abgabe des Arzneimittels (zumindest in Deutschland), 3) dass das Arzneimittel Gegenstand einer laufenden oder bald beginnenden Zulassungsprüfung sein muss, 4) dass das Programm für Patient*innengruppen konzipiert sein muss und 5) dass schwere Nebenwirkungen angezeigt werden müssen. Sind all diese Voraussetzungen für ein Härtefallprogramm erfüllt, zeigt das Pharmaunternehmen dieses Programm bei den zuständigen Behörden an. In Deutschland kommen dafür entweder das Bundesinstitut für Arzneimittel und Medizinprodukte oder das Paul-Ehrlich-Institut in Frage. Bleibt ein behördlicher Widerspruch aus, kann das Programm starten.

Pharmaunternehmen sind jedoch nicht zu einer Initiierung eines Compassionate Use-Programms verpflichtet (Huster et al. 2016, S. 57), weshalb viele erst nach medial aufgebautem Druck gestartet werden (Mackey und Schoenfeld 2016). Die Kriterien, ob, wem, was, wann und wieviel von einem Arzneimittel zur Verfügung gestellt wird, legt das Pharmaunternehmen selbst fest – denn für die Allokation von Arzneimitteln gibt es keinen Rechtsrahmen. Den zuständigen Behörden liegen nach eigener Anfrage auch keine Aufzeichnungen über die bisher erfolgten Allokationsmethoden vor.

### Onasemnogenum abeparvovecum

Das 2020 gestartete Programm um das Gentherapeutikum *Onasemnogenum abeparvovecum* erfuhr besondere mediale Aufregung (Chapman 2019; TreatSMA 2019; Ettel 2020; Kaulen 2020). *Onasemnogenum abeparvovecum* ist ein nur einmalig zu applizierendes Gentherapeutikum zur kurativen Therapie von Spinaler Muskelatrophie (SMA) bei Kindern.^1^ Es schleust künstlich bearbeitete Adenoviren als Vektoren in humane Zellen ein, die deren Gencodierung verändern und so das pathognomonisch fehlende Gen *SMN1* ersetzen (Vill et al. 2017). Dadurch sollen genügend Proteine für die bei dieser Krankheit progressiv absterbenden Motoneurone zum Überleben erzeugt werden, wodurch der Krankheitsverlauf gestoppt wird (Vill et al. 2017). Lange gab es für die Spinale Muskelatrophie gar kein Medikament (Borell et al. 2015). Der 2016 in den USA bzw. 2017 in Europa zugelassene Wirkstoff *Nusinersen*^2^ erwies sich nach anfänglicher Zeit als mit deutlichen Einschränkungen behaftet, weil dieser in regelmäßigen Intervallen ins Nervenwasser gespritzt werden muss. Nach Versteifungsoperationen im Jugendalter ist die Applikation meist nur mehr unter computertomografischer Lokalisation möglich, was mit einer großen Strahlenbelastung einhergeht.

Grund für die Aufregung um *Onasemnogenum abeparvovecum* war die vorgeschlagene, global organisierte Allokationsmethode in Form einer Lotterie, welche ein Novum in der Allokation bei Compassionate Use-Programmen darstellte (Sucker-Sket 2019; BeNeLuxA Initiative 2020). Das herstellende Pharmaunternehmen begründete diesen Schritt mit der notwendigen Limitierung auf 100 Dosen weltweit pro Jahr, da Staaten, wo die Zulassung erfolgt war (d. h., zum damaligen Zeitpunkt nur in den USA) bzw. bevorstünde, Priorität bei der Versorgung genossen. Aus Kapazitätsgründen konnten zudem nicht mehr Dosen des neuartigen Gentherapeutikums produziert werden (AveXis 2019). Die Allokationsmethode sei durch ein eigens gegründetes Ethikkomitee vorgeschlagen worden und sollte von einer beauftragten Drittfirma in verblindeter Form durchgeführt werden (AveXis 2019). Das Compassionate Use-Programm sollte solange in Kraft sein, bis *Onasemnogenum abeparvovecum* überall zugelassen wurde. Es startete weltweit im Januar/Februar 2020 und lief in Deutschland bis zum Juli 2020, als die Zulassung des Arzneimittels in Europa erfolgte und es dadurch per Gesetz beendet wurde. Aufgrund von fehlenden Registern ist unklar, ob das Programm in anderen Staaten noch weiter läuft.

## Ethische Konflikte

Im Rahmen der medizinethischen Debatte um Compassionate Use-Programme treffen eine Vielzahl an Aspekten auf unterschiedlichen Ebenen aufeinander (Borysowski et al. 2017). Es geht um die Abwägung eines potenziellen Benefits und eines möglichen Risikos bei der Anwendung (noch) nicht zugelassener Arzneimittel aus der Sicht von informierten Patient*innen, doch genauso um das wirtschaftliche und womöglich existenzielle Risiko durch eine gefährdete Zulassung aufgrund von Arzneimittelanwendung außerhalb klinischer Studien aus der Sicht der Pharmaunternehmen, falls im Rahmen eines Compassionate Use-Programms neue Fragen oder Komplikationen auftreten sollten. Es geht um den Einfluss der Dauer von Zulassungsverfahren per se, welche zwar in den letzten Jahren durch verschiedene Beschleunigungsverfahren teilweise verkürzt wurden (Kantor und Haga 2021), jedoch eine gewisse Zeit benötigen, um einen Sicherheitsmechanismus im Zulassungsverfahren gewährleisten zu können.^3^ Es geht um die klinische Prüfung von Arzneimitteln zur Sicherstellung der Anwendung für zukünftige Patient*innen, doch genauso um ein Recht auf Ausschöpfen aller erdenklichen Möglichkeiten bei einer ansonsten alternativlos zur schweren Behinderung oder zum Tod führenden Erkrankung von jetzigen Patient*innen. Es geht um Forschungsfragen, ab wann die Zahl von Proband*innen mit seltenen Erkrankungen eigentlich suffizient genug für eine klinische Aussage ist, doch auch darum, Forschung an seltenen Erkrankungen zu fördern anstatt zu bremsen. Es geht um soziale Verantwortung der Gesellschaft in Form von monetären Mitteln und Bereitstellung von Einrichtungen, doch auch um intrapersonelle Konflikte auf Seiten der Behandler*innen und Hersteller*innen. Es geht bei eingangs erwähntem Beispiel zusätzlich um Evidenz bei Therapien bei Kindern, doch auch um Rationierungen, welche bei neuartigen Behandlungsoptionen wie Gentherapeutika nur schwer bis gar nicht von extern überprüfbar sind.

Und es geht um Gerechtigkeit. Das geplante Allokationsverfahren per Los wurde vielfach als ungerecht kritisiert. Das Pharmaunternehmen würde sich „aus der Verantwortung stehlen“ (Sucker-Sket 2019), es entstünde großer Druck auf alle Beteiligten (Ettel 2020), Betroffene dieser vulnerablen Kohorte könnten sich nun als Kontrahent*innen wahrnehmen (Dyer 2020), der Empfängerkreis sei nicht objektiv durch klare Kriterien bestimmt (DIVI – Deutsche Interdisziplinäre Vereinigung für Intensiv- und Notfallmedizin 2020) und die Bedürftigkeitsprüfung durch die fehlende Transparenz sei nicht garantiert (Deutsches Ärzteblatt 2020). Losverfahren würden individuelle Nuancen bei der Vergabe missachten (Voigt und Freuler 2020). Das hohe Maß an Unsicherheit und die Intransparenz des Verfahrens seien inakzeptabel (Dyer 2020). Einige europäische Gesundheitsministerien gaben in einer gemeinsamen Aussendung starke Vorbehalte gegenüber dem Losverfahren an, da sich eine solche Allokationsmethode erheblich von früheren Programmen unterscheide (BeNeLuxA Initiative 2020). Das Schicksal von Patient*innen einer Lotterie gleichzusetzen, „ließe Menschenwürde und moralische Werte außer Sicht geraten“ (BeNeLuxA Initiative 2020). Lotterien seien „von Natur aus eine Form von Spiel“ und daher im Gesundheitssystem bei den Sorgen der Menschen absolut der falsche Ansatz (Dyer 2020). Trotzdem wurde das Programm in den meisten Staaten genehmigt, meist in Ermangelung praktikablerer Alternativen (Maybaum 2020).

Daher ist Ziel dieser Arbeit, anhand des gegenständlichen Beispiels um *Onasemnogenum abeparvovecum* ein allgemeines Modell abzuleiten, welches bei globaler Allokation von begrenzt verfügbaren und nicht teilbaren Gütern wie Arzneimitteln im Rahmen von Compassionate Use-Programmen bei Kindern Anwendung finden kann. Dazu werden mögliche Allokationskriterien vorgestellt, betreffend deren globaler Anwendbarkeit bei einmalig zu applizierenden Arzneimitteln diskutiert und nicht ausgeschlossene Kriterien anschließend hierarchisch geordnet. Da die globale Allokation per Losverfahren ein Novum ist, gibt es in der Literatur keine Handlungsempfehlungen für die Allokation von einmalig zu applizierenden Arzneimitteln für Kinder bei Compassionate Use-Programmen.

## Allokationskriterien

Viele verschiedene Allokationsprinzipien wurden bei der Verteilung von medizinischen Ressourcen bereits alleinig oder kombiniert angewandt oder werden es täglich. Frau/man denke an kleinere tägliche Unterschiede in der Terminvergabe („First-Come-First-Served“), oder der unterschiedlich aufgebrachten Zeit zur Behandlung (Dringlichkeit), oder an größere Diskussionen um die Zuweisung an Organersatzverfahren wie Dialysegeräte in früheren Mangelzeiten, der Zuteilung von intensivmedizinischen Kapazitäten in Pandemiezeiten oder der Abwägung zwischen Erfolgsaussicht und Dringlichkeit in der Allokation von Transplantaten. Zur Verteilung von unteilbaren, begrenzt verfügbaren, medizinischen Gütern gibt es eine Reihe an verschiedenen Prinzipien, nach welchen Güter verteilt werden können. Persad et al. (2009) teilten diese in vier Kategorien ein (Tab. 1).

**Table 1:** 

Gleichbehandlung	Lotterie „First-Come-First-Served“
Schadensvermeidung	Kränkste Patient*innen zuerst Jüngste Patient*innen zuerst
Benefitmaximierung	Anzahl der geretteten Leben Anzahl der geretteten Lebensjahre
Förderung und Belohnung des sozialen Nutzens	Werte für die Zukunft Werte aus der Vergangenheit

Ein weiteres Verteilungsprinzip in akuten medizinischen Nöten könnte die Bevorzugung von Patient*innen sein, welche bereits an klinischen Studien teilgenommen haben (Emanuel et al. 2020). Die Priorisierung von Personen, die an Forschungsarbeiten zum Nachweis der Sicherheit und Wirksamkeit von Therapien teilnahmen, könnte deren Risikoübernahme belohnen und weitere Personen ermutigen, an klinischen Studien für zukünftige Patient*innen teilzunehmen (Emanuel et al. 2020).

Darüber hinaus besteht die Möglichkeit, medizinische Güter den Meistbietenden zu vermachen, sie also im kapitalistischen Sinn zu verkaufen oder zu versteigern.

### Anwendung dieser Prinzipien für Onasemnogenum abeparvovecum

Ein „First-Come-First-Served“-Verteilungsprinzip von 100 Dosen *Onasemnogenum abeparvovecum* würde ein zeitliches Wettrennen um die Antragstellung verursachen. Wer sich am schnellsten meldet, bekäme den Zuspruch und damit nach erfolgtem Transport auch das Medikament. Wie würde so eine Zuteilungsform in der Praxis aussehen? Schon der Zeitpunkt des „Öffnens“ des Zuteilungsverfahrens wäre komplex und würde Bevorzugungen und Benachteiligungen generieren. Gilt das Eintreffen des Antrags bei dem Hersteller als Zeitpunkt der Listung? Dann haben Patient*innen bzw. Angehörige, die örtlich weiter von der ausgewählten Entscheidungsstelle zur Bestimmung der Verteilung entfernt wohnen, deshalb einen Nachteil. Wenn die Antragstellung aus der Ferne erfolgen kann, wie wäre dies geregelt? Auf dem analogen, postalischen Weg hängt die Reihenfolge des Eintreffens der Anträge maßgeblich davon ab, wie schnell und zuverlässig das mit der Zustellung beauftragte Unternehmen funktioniert. Ist die Beantragung wiederum online möglich, werden all jene benachteiligt, denen ein Onlinezugang nicht zur Verfügung steht, er just in dem Moment nicht funktioniert oder dieser schlicht nicht beherrscht wird. Die korrumptive Anfälligkeit dieses Verteilungsprinzips (Persad et al. 2009) könnte in Richtung jener Bewerber*innen ausschlagen, die einen direkten Zugang zum Hersteller besitzen – sei es durch örtliche Nähe, durch Kontakte oder durch bisherige Zusammenarbeit in anderen Projekten. Patient*innen von Ärzt*innen, die mehr mit Novartis zu tun haben und vielleicht sogar Drittmittel von Novartis beziehen, könnten dadurch in einen nicht mehr wettzumachenden Vorteil gegenüber anderen Patient*innen kommen.

Werden die kränksten Patient*innen bei der Allokation bevorzugt, stellen sich viele diagnostische Fragen bezüglich der Dringlichkeit. Wie definiert man diese im Fall einer erwartet kurativen, nur einmalig zu applizierenden Therapie? Dafür kommen mehrere Kriterien in Frage: Aus der Sicht der/des Einzelnen kann argumentiert werden, dass die Dringlichkeit für alle gleich ist, da es um eine Kuration (bzw. einen Stopp der weiteren Progression) der Erkrankung geht und jeder Zeitverlust mit einer Verschlechterung einhergeht. Andererseits kann über eine größere Kohorte Dringlichkeit in unterschiedlichem Ausmaß definiert werden. Dieses unterschiedliche Ausmaß – dass für eine*n Patient*in eine größere Dringlichkeit darstellt und sie/er daher bevorzugt werden sollte, während für eine*n andere*n Patient*in aufgrund von weniger großer Dringlichkeit deshalb ein Nachrang entsteht – bedarf einer Ausformulierung an Gewichtungen, damit zu einer spezifischen Reihung gefunden werden kann. Dafür kommen Symptome (also der Phänotyp der Erkrankung) und/oder der Erbtyp der Erkrankung (Genotyp) in Frage. Diesbezüglich besteht allerdings keinerlei Evidenz, wie welche Faktoren zu erheben und zu gewichten wären. Daher muss zuerst ein Diskurs zu einem Konsens unter Expert*innen führen, die die Gewichtung von phänotypischen Symptomen und Funktionen sowie des Genotyps der Erkrankung festlegen. Dies könnte allerdings sehr oder zu lange dauern, was in Anbetracht des Zeitdrucks kontraproduktiv ist und durch eine globale Verteilung sicher nicht vereinfacht wird. Zumal ein Konsens unter Expert*innen keinesfalls garantiert ist, da durch den neuen Therapieansatz der Gentherapie bisher keinerlei Evidenz vorhanden ist. Selbst wenn Kriterien erstellt worden sind, die eine Gewichtung ermöglichen, wie gestaltet sich dann die Erhebung dieser Kriterien von allen Bewerber*innen? Ist der Genotyp zwar diagnostisch festlegbar, so liegen global gesehen jedoch nicht überall alle Möglichkeiten der genetischen Diagnostik vor. Diese Proben sind vermutlich auch nicht „versendbar“ oder auf Abruf transportabel, und können aufgrund technischer Einschränkung auch nicht von jedem Ort der Welt in ein zu ermittelndes Zentrallabor gebracht werden, um anhand einer genetischen Klassifizierung (wie beispielsweise der Anzahl kompensatorischer Gene) eine Reihung vorzunehmen. Der Phänotyp hingegen kann zwar beschrieben oder ermittelt werden, allerdings fehlen diesbezüglich geeichte und standardisierte Protokolle. Wiederum ist die knappe oder vielerorts fehlende Ressource der Diagnostik zu bedenken. Bei den Studien zur SMA-Forschung hat sich der CHOP INTEND-Score der Kinderklinik Philadelphia zur Erfassung der neuromuskulären Beschwerden bewährt (Glanzman et al. 2010), ob dieser jedoch alle Parameter zur Erstellung einer Dringlichkeitsreihung erfasst, ist unklar. Möglicherweise werden Patient*innen absichtlich schlechter eingestuft, um deren Zuteilungschancen zu erhöhen. Die Gesetzgeber*innen müssten zuvorkommen und eine absichtliche Schlechter-Stellung von Patient*innen, ähnlich wie beim Transplantationsskandal in den 2010er Jahren, durch deren behandelnde Ärzt*innen (zum eigentlich beabsichtigen Wohl ihrer Patient*innen) in Strafe stellen. Hierfür einen globalen Konsens zu finden, könnte äußerst schwierig sein. Ein Leichteres wäre die Definition von Dringlichkeit alleine dadurch, ob alternative Therapieoptionen existieren. Das heißt, wo beispielsweise andere zugelassene Medikamente zur Behandlung der SMA wie *Nusinersen* zugelassen sind. Damit wären Staaten wie Deutschland bei der Verteilung auszuschließen – obwohl dort wahrscheinlich eine vermeintlich größere Expertise (durch Vorerfahrungen) und mehr Ressourcen des Gesundheitssystems (durch spezialisierte Zentren für SMA) für eine Behandlung mit *Onasemnogenum abeparvovecum* zur Verfügung stünden. Zudem fehlen in nicht-wohlhabenden Staaten die Möglichkeiten der genotypischen und teilweise sogar der phänotypischen Diagnostik gänzlich.

Auch das Verteilungsprinzip zugunsten der jüngsten Patient*innen wird durch die gegebenen Ressourcen eines nationalen Gesundheitssystems beeinflusst. Während wohlhabende Staaten dazu in der Lage sind, intrauterine genetische Diagnostik durchzuführen, kann in ärmeren Staaten die Diagnose erst viel später gestellt werden. Der Faktor Zeit spielt bei dieser Verteilung ebenso mit, da evident ist, dass sich die Fähigkeiten der Patient*innen und die Prognose linear und irreversibel mit dem Fortschritt der Erkrankung verschlechtern. Gibt man die 100 vorhandenen Dosen an die bei Ausgabe jeweils jüngsten, wahrscheinlich gerade erst geborenen Kinder mit gendiagnostizierter SMA ab, würden diese 100 Kinder symptomfrei leben können, ohne dass jemals eine andere Therapieindikation bestehen würde. Andererseits könnte die Penetranz der Erkrankung deutlich milder verlaufen, als es die genetische Diagnostik prognostizieren würde. Dabei steht eine Zulassung des Medikaments ja in Bälde im Raum, wodurch zumindest das Argument des zeitlichen Aufschubs bei temporär begrenzten Ressourcen Geltung finden könnte. Wird der Zuteilungsprozess zeitlich determiniert, könnte es sogar dazu führen, dass Geburten absichtlich verzögert oder vorgezogen werden, nur um bei der Zuteilung zugunsten der Jüngeren den Zuschlag zu erhalten.

Bezüglich der Benefitmaximierung steht bei der Vergabe von *Onasemnogenum abeparvovecum* die limitierte und unteilbare Dosis im Vordergrund. 100 Kinder können geheilt werden, alle anderen nicht (zumindest nicht durch diese Therapie) – es entsteht eine binäre Entscheidungssituation. Entscheidet man nun utilitaristisch nach der Anzahl der geretteten Leben, ergibt es primär keinen Unterschied, welche Kinder die Dosen bekommen würden und welche nicht. Sekundär wäre allerdings zu bedenken, dass manche Kinder aufgrund vorhandener Therapiealternativen und unterschiedlicher Krankheitsausprägung weiter behandelt würden und länger leben könnten, andere jedoch nicht. Die Anzahl der geretteten Lebensjahre dürften sich im Wesentlichen dennoch kaum unterscheiden, da bei *Onasemnogenum abeparvovecum* ausschließlich von Kindern gesprochen wird. Dadurch ist es quasi unbedeutend, ob ein 3‑jähriges oder ein 4‑jähriges Kind zur Verteilung bereitsteht, da jedes der Kinder das gesamte Leben noch vor sich hat. Ein unterscheidender Faktor könnte jedoch die Erfolgsaussicht sein, die unterschiedlich ausgeprägt sein kann, je nachdem, welcher Geno‑/Phänotyp vorliegt (siehe dazu Diskussionspunkt Dringlichkeit) und wie die gesamte Einbindung in die Behandlungsumgebung ist. Diese Einbindung hängt allerdings neben der lokal gegebenen Infrastruktur maßgeblich von den, in der Pädiatrie typischerweise durch die Dreieckskonstellation involvierten Angehörigen ab (ob diese die Compliance und die Bewältigungsfähigkeit der Erkrankung ihres Kindes besitzen) und nicht von dem erkrankten Kind selbst. Inkludiert man den Gedanken, dass bei einer positiven Zuteilung das Kind kuriert wird und ohne weitere Hilfe auskommen kann, müsste man schlussfolgern, dass es zur Minimierung der Krankheitslast am fairsten wäre, das Medikament denjenigen zuzuteilen, deren Angehörige nicht im Stande sind, die Therapien adäquat zu begleiten, die Krankheitslast zu bewältigen und die am weitesten entfernt von einem Therapiezentrum liegen oder am schlechtesten angebunden sind. Dies erscheint insofern fragwürdig, als dass ggf. Incompliance bevorzugt bewertet werden würde. Abgesehen von den Analogien zu den Diskussionspunkten des Prinzips Dringlichkeit könnte die Erfolgsaussicht bei Zuteilung einer kurativen Maßnahme als „für alle gleich“ postuliert werden: Einer Heilung (oder zumindest einem Stopp der weiteren Verschlechterung) bei Zuteilung und einem Beibehalten des „Status quo“ und damit – je nach Anbindung in die Behandlungsumgebung – eine mehr oder weniger progrediente Verlaufsform bei Nicht-Zuteilung. Es muss aber davon ausgegangen werden, dass zwar der Krankheitsverlauf gestoppt, nicht aber bereits verloren gegangene Fähigkeiten wieder erworben werden können. Dazu gibt es bis dato keine Evidenz, daher beruht dies nur auf einer Annahme. Dies wiederum führt dazu, dass die Erfolgsaussicht doch nicht als „für alle gleich“ anzunehmen ist, sondern sich in der Ausprägung der Erkrankung widerspiegelt. Je weiter diese Progression bis zur Zuteilung bzw. Applikation des Medikaments fortgeschritten ist, desto schlechter die Erfolgsaussicht. In Anbetracht dieser Annahme wären bei progredientem Verlauf die jüngsten Patient*innen zu bevorzugen, während ältere Kinder aufgrund des bereits fortgeschritteneren Stadiums bei einer Zuteilung nach Erfolgsaussicht nicht mehr zum Zug kommen. Bei dieser Definition der Erfolgsaussicht, die mit dem Alter korreliert, würde dann wiederum das Prinzip zugunsten der Jüngeren zuerst in Aussicht gestellt, welches aufgrund der möglichen Verzögerungen oder Vorziehungen von Geburten aber abzulehnen ist.

Die instrumentelle Wertzuweisung von Kindern anhand ihrer Vergangenheit oder Zukunft zu beurteilen, erscheint unmoralisch. Eine verantwortungsbasierte Zuweisung, wie beispielsweise Influenza-Impfstoffe an jene zu verteilen, die bei der Herstellung mitwirken, ist bei Kindern obsolet. Kinder konnten noch gar keine Möglichkeit entwickeln, um selbstständig am sozialen Leben teilzunehmen. Eine Übernahme des sozialen Nutzens der jeweiligen Angehörigen erscheint unzulässig.

Als diskutabel erscheint das Prinzip der Bevorzugung durch die Teilnahme an klinischen Studien. Damit ist nicht gemeint, dass Compassionate Use-Programme nicht direkt einem Erkenntnisgewinn dienen – was sie nicht dürfen, da sich diese per Definition von klinischen Studien unterscheiden müssen – sondern ob Patient*innen bei früheren Studien dazu bereit waren, an der Entwicklung des Arzneimittels teilzunehmen. Auch dies bedingt jedoch die Anbindung an ein entsprechend adäquat ausgestattetes Behandlungsteam sowie die zu erfüllenden Einschlusskriterien, was die Teilnahme an der klinischen Forschung erst ermöglichen hätte können. Daher könnte die Teilnahmebereitschaft in eine Allokationsentscheidung einbezogen werden, auch wenn grundsätzlich hervorzuheben ist, dass Teilnahme an Forschung gemäß der Definition von Helsinki freiwillig zu erfolgen hat und hier kein Zwang für einen eventuell späteren Benefit bei der Entscheidung zur Teilnahme mitwirken darf.

Ein Verkaufen oder Versteigern von Arzneimitteln erscheint unmoralisch. Da Medizin grundsätzlich als keine Dienstleistung und Gesundheit damit als nicht kaufbar gesehen wird, bleibt dieses Verteilungsprinzip außen vor.

### Hierarchische Ordnung der verbliebenen Prinzipien

**Figure 2:**
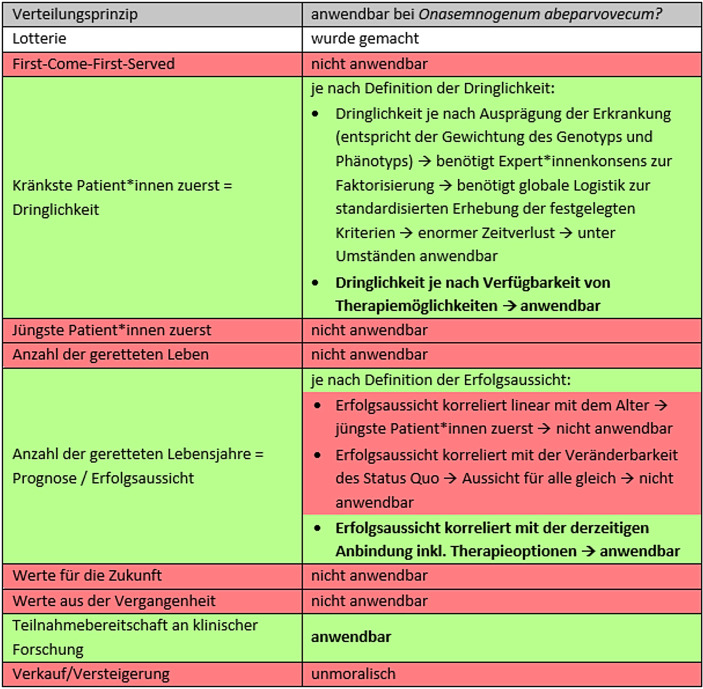

Abb. 2 fasst die genannten Prinzipien zur Verteilung von unteilbaren, begrenzt verfügbaren medizinischen Gütern am Beispiel des Compassionate Use-Programms für *Onasemnogenum abeparvovecum* zusammen. Von zehn möglichen Verteilungsprinzipien verbleiben aufgrund des Krankheitsbilds der SMA mit progredientem Verlauf durch Verlust der körperlichen Funktionen drei Prinzipien als Alternative zur Lotterie (grün dargestellt). Die Verteilung nach den Prinzipien Dringlichkeit und Erfolgsaussicht ist dabei je nach deren Definition unter Umständen mit einzubeziehen.

Da ein Losverfahren bei der Erstellung einer hierarchischen Ordnung hintenangestellt wird – andernfalls würde es alle anderen Prinzipien ad absurdum führen, wenn es nicht als letztes gereiht wäre – verbleiben lediglich drei Prinzipien in der Diskussion.

Ohne nennenswerte Abstriche besteht nur das Prinzip der Teilnahmebereitschaft an früher stattgefundenen klinischen Studien, welches damit als einziges für die Reihung an erster Position der Ordnung nominabel erscheint. Grundsätzlich bestehen auch hier Nachteile wie die Erforderlichkeit eines adäquaten Anschlusses an ein Behandlungszentrum, darum ist explizit die Teilnahmebereitschaft und nicht die Teilnahme zu reflektieren. Zu bedenken ist hier die kurative Wirkung des Arzneimittels: All jene Patient*innen aus vorherigen klinischen Studien, die das Arzneimittel bereits erhalten haben, benötigen es aufgrund der Einmalgabe nicht mehr. Damit verbleiben lediglich jene Kinder zur Verteilung, die das Arzneimittel entweder gar nicht (da in einer randomisierten Studie der Kontrollgruppe zugeordnet) oder in zu geringerer Dosis (da in einer Dosisfindungsstudie) erhalten haben. Aufgrund der niedrigen Inzidenz der Erkrankung („orphan disease“) waren die Studienkohorten sehr klein, daher werden also nur wenige Patient*innen durch das Prinzip der bisherigen Teilnahmebereitschaft an klinischen Studien zur Entwicklung und Erforschung von *Onasemnogenum abeparvovecum *erfasst. Dazu kommt, dass diese Studien in den USA durchgeführt wurden (Finkel et al. 2017). Die bei dieser Zuteilungsform vorrangig gewählten Patient*innen haben dementsprechend schon Zugang zum Medikament durch dessen Zulassung in den USA erhalten. Daher scheidet das Prinzip in der Praxis als mögliches Zuteilungskriterium aus.

Nachfolgend rangieren mit Dringlichkeit und Erfolgsaussicht zwei diskutable Prinzipien. Beide sind jeweils unterschiedlich definierbar. Bei der Dringlichkeit gibt es aufgrund des bestehenden Zeitdrucks nur limitierte Möglichkeiten zur Faktorisierung von noch zu bestimmenden Gewichtungen sowie deren standardisierte Umsetzung auf globaler Ebene. Damit verbleibt die Definition der Dringlichkeit je nach Verfügbarkeit von Therapiemöglichkeiten. Bei der Erfolgsaussicht scheiden die Alterskorrelation sowie die Veränderbarkeit des Status Quo aus, daher verbleibt die Anbindung an ein Therapiezentrum mit Behandlungsoptionen. Beide Prinzipien, Dringlichkeit und Erfolgsaussicht, sind also mit der Problematik der globalen Verteilungsfrage verknüpft.

Neben den unterschiedlichen Ressourcen bestehen zwischen den einzelnen Staaten keine Abkommen, die eine unabsichtliche oder absichtliche Besser- oder Schlechterstellung von Patient*innen in einem „Wettbewerb“ um die höchste Dringlichkeitsstufe regulieren. Daher kann – nach erfolgter Festlegung von Kriterien durch einen Expert*innenkonsens – durch die Delegation an jeweils national verantwortliche Personen nicht verhindert werden, dass es zur Problematik der Klassifizierung zwischen Patient*innen und in Folge zwischen Staaten kommt. Um dieser Schwäche und Korruptionsanfälligkeit von national Delegiert*innen vorzubeugen, müsste ein a‑priori festgelegtes Delegiert*innenteam zu jedem einzelnen ansuchenden Kind reisen und die zuvor festgelegten Parameter selbst erheben bzw. überprüfen. Dieses Verfahren dauert angesichts der globalen Allokation sehr lange und steht im Widerspruch zu einer möglichst rasch durchzuführenden Zuteilung (und der in Aussicht stehenden Marktzulassung zum Zeitpunkt der Beantragung des Compassionate Use-Programms). Diese Argumente treffen auf den hier diskutierten Präzedenzfall von *Onasemnogenum abeparvovecum* zu; Ziel im Sinne eines Gedankenexperiments ist jedoch, ein allgemein gültiges Modell zur Erstellung von Dringlichkeitsstufen für solche Krankheiten und zukünftige Therapieoptionen zu finden. Da bei anderen Erkrankungen wiederum andere Scores zur Gewichtung von Krankheitsausprägungen und Schweregraden vorhanden sein können (oder noch zu erstellen sind), ist diese Überlegung zur Klassifizierung von Genotyp und Phänotyp zur Beurteilung der Dringlichkeit von potenziell heilbaren Medikamenten bei Einmalgabe nicht verallgemeinerbar.

Somit verbleiben die nationale Verfügbarkeit von Therapiemöglichkeiten (durch bereits erteilte Zulassung von anderen Medikamenten wie beispielsweise *Nusinersen*) und die individuelle Anbindung von Patient*innen an Therapiezentren (welche diese Behandlung und andere symptomatische Therapien durchführen können) als Surrogate der Dringlichkeit und Erfolgsaussicht übrig. Beide sind jedoch reziprok anzuwenden, was bedeutet: je schlechter die Anbindung an ein Therapiezentrum und je weniger supportive Therapieoptionen vorhanden, desto schlechter die Erfolgsaussicht und Dringlichkeit und desto höher der Nutzen bei der nur einmalig zu applizierenden Gabe eines kurativen Medikaments.

Auf globaler Ebene lässt sich also schlussfolgern:

i. Zunächst sollten all jene Patient*innen den Vorzug zur Erteilung des Arzneimittels bekommen, die zuvor zu einer Teilnahme an klinischen Studien zur Erforschung dieses Arzneimittels bereit waren, es aber aus studientechnischen Gründen nicht erhalten konnten (Prinzip der Bevorzugung für die Teilnahmebereitschaft an der Forschung).
ii. Danach sind all jene Patient*innen zu bevorzugen, die keinen Zugriff auf bestehende Therapieoptionen (zur Verlangsamung der Krankheitsprogression oder zumindest zur symptomatischen Behandlung) besitzen, weil an deren Aufenthaltsort keine Zulassung erteilt wurde und keine therapeutische Infrastruktur vorhanden ist (Kombination der Prinzipien der Dringlichkeit und der Erfolgsaussicht).

Die Lotterie findet in der lexikalischen Ordnung letztgereiht Verwendung, wenn die Anzahl der in i) und ii) zu erfüllenden Ansprüche zu groß wird und diese gleichwertig sind. Die Lotterie ist allerdings nur zu berücksichtigen, wenn nach Mobilisierung aller Vernunft- und Verhandlungskräfte kein weiteres Verteilungsprinzip mehr zur Verfügung steht:

iii. Übersteigt die Anzahl der Inanspruchnahmen nach Anwendung von i) und ii) die vorhandenen Dosen an Medikamenten, so ist bei Gleichwertigkeit das Los anzuwenden (Prinzip des Egalitarismus).

Hinsichtlich *Onasemnogenum abeparvovecum* bedeutet dies global gesehen, dass i) bei der Verteilung übersprungen wird, da die Zulassungsstudien nur in den USA durchgeführt wurden und die Patient*innen, die für i) in Frage kommen, bereits regulär aufgrund der erfolgten Zulassung durch die FDA Zugang zu *Onasemnogenum abeparvovecum* besitzen. Für ii) würden all jene Staaten ausscheiden, bei denen eine im globalen Vergleich adäquate medizinische Versorgung vorhanden ist (gute Anbindung an Therapiezentren, geeignete symptomatische Behandlung) und *Nusinersen* (zur spezifischen Therapie) zugelassen ist. Selbst wenn eine Therapie mit *Nusinersen* im Verlauf nicht mehr möglich ist (z. B. durch Unverträglichkeit), schließt die vorhandene medizinische Infrastruktur Patient*innen aus diesen Staaten aus, da supportive Therapien (Physiotherapie, Ergotherapie, Rollstuhltraining, Beatmungsmöglichkeiten) weiterhin angewendet werden können. Der europäische Raum käme also bei der Verteilung von einem globalen Compassionate Use-Programm mit nur 100 verfügbaren Dosen an *Onasemnogenum abeparvovecum* wohl nicht zum Zug. Stattdessen würden die Dosen nur an jene Staaten verteilt werden, die diese Kriterien nicht erfüllen. Sind i) und nachfolgend ii) ausgeschöpft, kann gemäß der lexikalischen Ordnung auf iii) zurückgegriffen werden, wenn die Anzahl an Anfragen die Anzahl der Dosierungen übersteigt.

## Beurteilung

Aus der Ableitung potenzieller Allokationsprinzipien sowie unter Berücksichtigung global unterschiedlicher Therapieoptionen wurde eine hierarchische Ordnung von möglichen Allokationskriterien unter dem Gesichtspunkt des Compassionate Use-Programms von *Onasemnogenum abeparvovecum* diskutiert. Daraus resultierte eine Kombination der Prinzipien der Teilnahmebereitschaft an Forschung, Dringlichkeit und Erfolgssausicht als Modell für global laufende Compassionate Use-Programme bei einmalig zu applizierenden Arzneimitteln bei Kindern. Eine Lotterie imponiert nur als letzte Option, wenn nach Mobilisierung aller Vernunft- und Verhandlungskräfte kein weiteres Verteilungsprinzip mehr zur Verfügung steht.

Es hätten also durchaus Kriterien gefunden werden können, die einem Losverfahren vorgezogen hätten würden müssen. Der Umstand, dass durch die geringe Anzahl der Proband*innen an klinischen Studien kaum Evidenz für einen Therapieerfolg oder -misserfolg besteht, verzeiht nicht die Tatsache, universellere Kriterien wie vorhandene Therapieoptionen in verschiedenen Staaten zu berücksichtigen. Alle Patient*innen in einen Lostopf zu werfen, ignoriert diese einfach zu erhebenden und nicht mit zeitlichem Aufschub verbundenen Parameter global unterschiedlich verteilter Merkmale gänzlich. Zudem hätte bereits in früheren Studienphasen nach medizinischen Befunden Ausschau gehalten werden müssen, welche eine eventuell später notwendige Allokation im Fall eines Compassionate Use-Programms medizinisch und damit auch moralisch begründbar gemacht hätte.

Welches Verteilungsverfahren auch immer bei knappen medizinischen Ressourcen gewählt wird, es muss moralisch relevante Werte anerkennen und legitim sein (Persad et al. 2009). Diese Legitimität erfordert, dass die gewählte Methodik als gerecht und fair akzeptiert wird. Dazu benötigt es nicht nur Verfahrensgerechtigkeit, sondern auch Zuteilungsgerechtigkeit. Beides ist hinsichtlich der Diffizilität der globalen Allokation anspruchsvoll. Zur Vertrauensbildung bedarf es den Einbezug der Öffentlichkeit, denn die Verständlichkeit des Problems kann nur in einem Diskurs und durch Transparenz im gesamten Verfahren hergestellt werden. Um mit einem Kriterienkatalog eine gerechte Verteilung bewirken zu können, müssen die durchdachten Prinzipien in einem unparteiischen Prozess festgelegt werden. Dafür kann ein unabhängiges Gremium eingesetzt werden, was im geschilderten Beispiel auch gefordert wurde (Maybaum 2020; DIVI – Deutsche Interdisziplinäre Vereinigung für Intensiv- und Notfallmedizin 2020). Ein solches Gremium an Expert*innen könnte Entscheidungslasten erleichtern (Borysowski et al. 2017), und den Eindruck reduzieren, unternehmerischer Willkür ausgeliefert zu sein (Huster et al. 2016, S. 113). In einzelnen Staaten, wie z. B. Australien, ist dies bei nationalen Compassionate Use-Programmen verpflichtend (Borysowski et al. 2017).

Das bekannteste Gremium ist das „CompAC“ der New York University School of Medicine, welches 2015 auf Ansuchen eines Pharmaunternehmens ins Leben gerufen wurde (Caplan et al. 2018). In diesem Gremium wurde verblindet über die nationale Allokation eines nicht in ausreichender Menge vorhandenen Medikaments für ein Compassionate Use-Programm zur Therapie von Plasmazelltumoren bei Erwachsenen entschieden (Caplan et al. 2018). Die beratende Einheit berichtete von zufriedenstellenden Erfahrungen, schränkte jedoch auch ein, dass final kein „gutes Tool“ zur Allokation gefunden werden konnte – und es dieses womöglich auch gar nicht gibt (Caplan et al. 2018). Die angewendeten Kriterien seien schwer standardisierbar und können trotz Anwendung übergeordneter ethischer Prinzipien nicht als universeller Algorithmus dienen (Caplan et al. 2018).

Vor allem die Anwendung von einmalig zu applizierenden Arzneimitteln, die Zielgruppe Kinder und die globale Allokation potenzieren den Schwierigkeitsgrad der Suche nach einem bestmöglichen Allokationsprinzip. Eine Auslagerung der Verteilungsfrage an ein unabhängiges Gremium von Expert*innen könnte jedenfalls ein höheres Maß an Verfahrensgerechtigkeit garantieren – unabhängig davon, welche Allokationsmethode von einem solchen Gremium dann gewählt wird. Auch wenn ein globaler Konsens über die Notwendigkeit solcher Gremien schwierig erscheint, und nationale Verpflichtungen hierzu die Gefahr des Außen-vor-Gelassen-Werdens beinhalten, sollte gemeinsam mit anderen Staaten, die sich kritisch zur Allokation per Losverfahren über eine Drittfirma äußerten, versucht werden, eine größere Regulationshandhabe zu diesem Thema zu erreichen, um Willkür von Pharmaunternehmen vorzubeugen. Dazu braucht es die Verpflichtung, dass Allokationsverfahren an unabhängige und gemeinsam betriebene Gremien mit Entscheidungskompetenz delegiert werden müssen. Der hier vorgestellte Algorithmus zur globalen Allokation von einmalig zu applizierenden Arzneimitteln bei Kindern kann dabei eine Hilfe sein. Darüber hinaus benötigt es forcierte Anstrengungen, dass der Bedarf an Compassionate Use-Programmen sinkt, wie zum Beispiel durch schnellere Zulassungsverfahren.

## Footnotes

1 ’Die Spinale Muskelatrophie ist eine neurodegenerative Erkrankung, welche durch einen fortschreitenden Untergang von initial normal angelegten Nervenzellen kennzeichnet ist (Schaaf und Zschocke 2013, S. 274). Auf eine Phase der normalen psychomotorischen Entwicklung folgt charakteristisch ein „Knick“: Die Entwicklung stagniert auf dem bisher erreichten Niveau – bereits erlernte Fähigkeiten können jedoch auch wieder verloren gehen (Schaaf und Zschocke 2013, S. 274). Sie wird autosomal-rezessiv vererbt und betrifft das zweite Motoneuron in der Pyramidenbahn (Schaaf und Zschocke 2013, S. 279). Mit einer weltweiten Prävalenz von 1:11.000 ist sie die häufigste neurodegenerative Krankheit im Kindesalter (Vill et al. 2017) und betrifft Jungen im Verhältnis zu Mädchen circa doppelt so häufig. Je nach Ausprägung und Manifestation der Symptomatik werden verschiedene Schweregrade definiert (Borell et al. 2015). Bei Frühmanifestation (Typ 1) sind bereits nach kurzer Zeit keine Bewegungen gegen die Schwerkraft mehr möglich – Gedeihstörungen und Infekte sind die Folge, welche schnell lebensbedrohlich werden können. Dieser Typ kommt unter den Spinalen Muskelatrophien nicht nur am häufigsten vor (van der Ploeg 2017), sondern ist auch die häufigste genetisch bedingte Todesursache bei Säuglingen (Saffari et al. 2019). Typ 2‑Erkrankte stagnieren in ihrer Entwicklung gegen Ende des ersten Lebensjahres, behalten in der Regel aber die bereits erlernten Fähigkeiten wie Sitzen oder Kopfhalten bei. Während Typ 3 und Typ 4 durch ein späteres Einsetzen der Symptome und eine langsamere Progression gekennzeichnet sind und erkrankte Kinder das Erwachsenenalter gut erreichen, waren Typ 1 und Typ 2 bis vor kurzem mit einer eingeschränkten Lebenserwartung assoziiert. Neben der klinischen Einteilung wird die Diagnose molekulargenetisch gesichert. Für die Erkrankung ursächlich ist eine homozygote Deletion des *Survival of Motor Neuron 1* (*SMN1*)-Gens auf dem Chromosom 5q13 (Borell et al. 2015), wobei die Exons 7 und 8 fehlen (Schaaf und Zschocke 2013, S. 279ff.). Fehlt dieses *SMN1*-Gen, mangelt es an SMN-Proteinen, welche für das Überleben der Motoneurone sorgen. Das paraloge *SMN2*-Gen kann durch Selbstvermehrung die ursprüngliche Funktion von *SMN1* zumindest teilweise kompensieren (Borell et al. 2015), nicht aber die vollständige Funktion der Proteine wiederherstellen. Dementsprechend präsentiert sich der Phänotyp der Spinalen Muskelatrophie umso weniger ausgeprägt, je öfter die Kopie des *SMN2*-Gens vorliegt (Schaaf und Zschocke 2013, S. 280).

2 Nusinersen ist ein Antisense-Oligonukleotid – also eine kurze Nukleotidsequenz, die selektiv an Ribonukleinsäure bindet und damit das Gen *SMN2* vermehrt exprimieren lässt. Dadurch entsteht eine größere Menge an funktionsfähigen SMN-Proteinen (Vill et al. 2017), was bei allen SMA-Typen hilfreich ist, bei denen *SMN2* vorhanden ist. Das Medikament muss über eine Lumbalpunktion ins Hirnwasser („intrathekal“) appliziert werden, um direkt vor Ort an den Motoneuronen wirken zu können. Auf mehrere Applikationen zur initialen Aufsättigung folgt die regelmäßige Weiterverabreichung in bestimmten, pharmakogenetisch bedingten Intervallen (meist 3–4 Monate). Gerade der Applikationsweg und die nötigen Wiederholungen wurden jedoch als Hauptfaktoren für Nebenwirkungen wie Infektion, Toxizität oder komplikationsbehaftete Verabreichung ausgemacht (Vill et al. 2017). Speziell für ältere Erkrankte könnte die intrathekal notwendige Applikation durch zunehmende Skoliose oder nach Operationen an der Wirbelsäule zu deutlichen Problemen im Verlauf führen (Vill et al. 2017). Oft ist dafür eine Narkose nötig.

3 Die generelle Dauer von Zulassungsverfahren ist ein Hauptgrund für ethische Konflikte bei Compassionate Use-Programmen, da eine Vielzahl dieser Programme überhaupt erst dadurch in Betracht gezogen wird, weil das Zulassungsverfahren mindestens mehrere Monate in Anspruch nimmt (siehe Abb. 1). Probleme wie zum Beispiel Komplikationen, die im Rahmen eines Compassionate Use-Programms auftreten, können die Zulassung weiter verzögern oder gefährden (Meier 2016). Andererseits könnte das prinzipielle Beschleunigen eines Zulassungsverfahrens die Notwendigkeit von Compassionate Use-Programmen und damit hier angeführte moralische Dilemmata zumindest reduzieren. Abzuwägen sind hierbei selbstredend immer Sicherheitsaspekte, deren Prüfung nicht auf eine Minimalzeit reduzierbar rational erscheint. Obwohl verschiedene Pfade zu einer beschleunigten Zulassung von Arzneimitteln in den letzten Jahren vermehrt Anwendung gefunden haben (Kantor und Haga 2021), gibt es Daten über eine erhöhte Wahrscheinlichkeit für nachträgliche Warnungen bei diesen Arzneimitteln (Frank et al. 2014). Ein schnelleres Zulassungsverfahren könnte die Überprüfung von Qualität und Evidenz beeinträchtigt haben (Frank et al. 2014). Eine Reihe von Richtlinien adressiert diese Problematik (Naci et al. 2020), welche in anderen Untersuchungen jedoch deutlich niedrigere Zahlen ergab (Kantor und Haga 2021). Ob neuartige Verfahren wie der sogenannte „Rolling review“, die in der Zeit der Covid-19-Pandemie zur äußerst schnellen Zulassung von Vakzinen führten (Marinus et al. 2022), auch bei seltenen Erkrankungen zu einem positiven Effekt hinsichtlich der Reduktion der Notwendigkeit von Compassionate Use-Programmen führen könnten, ist noch unklar.
